# Relationship between obesity-related parameters and chronic kidney disease in middle-aged and elderly populations in Taiwan: A community-based study

**DOI:** 10.3389/fnut.2022.928910

**Published:** 2022-10-04

**Authors:** I-Ju Chen, Le-Tien Hsu, Ting-Wei Lin, Jau-Yuan Chen

**Affiliations:** ^1^Department of Family Medicine, Chang Gung Memorial Hospital, Taoyuan City, Taiwan; ^2^Department of Gynecology and Obstetrics, Chang Gung Memorial Hospital, Taoyuan City, Taiwan; ^3^Department of Laboratory Medicine, Chang Gung Memorial Hospital, Taoyuan City, Taiwan; ^4^College of Medicine, Chang Gung University, Taoyuan City, Taiwan

**Keywords:** chronic kidney disease, triglyceride to high-density lipoprotein-cholesterol ratio, obesity, middle-aged and elderly, dyslipidemia

## Abstract

Globally, obesity is a major health problem and can markedly increase the risk of various diseases, including type 2 diabetes mellitus, hypertension (HTN), dyslipidemia, and chronic kidney disease (CKD). The association of obesity-related parameters, such as lipid parameters and their ratio, with CKD in clinical settings is not well understood. This study aimed to investigate the association of obesity-related parameters with CKD in the middle-aged and elderly population in Taiwan. This cross-sectional, community-based study recruited 400 participants (141 males and 259 females) aged 50 years or over from a community health promotion project at the Linkou Chang Gung Memorial Hospital (Guishan District, Taoyuan City) in 2014. Each participant completed a questionnaire including personal information and medical history during a face-to-face interview. Laboratory data were obtained from blood and urine sampling. The data were analyzed using *t*-test, chi-square test, Pearson's correlation test, multivariate logistic regression, and receiver operating characteristic (ROC) analysis. A total of 81 participants were identified as having CKD [estimated glomerular filtration rate (eGFR) < 60 ml/min/1.73 m^2^ or urine albumin/creatinine ratio ≥30 mg/g], and their mean triglyceride/high-density lipoprotein cholesterol (TG/HDL-C) ratio was 3.37 ± 2.72. The mean TG/HDL-C ratio of the 319 participants without CKD was 2.35 ± 1.66. After adjusting for age, TG/HDL-C was significantly positively correlated with blood pressure, body mass index, waist circumference, and fasting plasma glucose but not low-density lipoprotein cholesterol. There was a negative correlation between TG/HDL-C and eGFR. Multiple logistic regression model analysis showed that TG/HDL-C was still significantly associated with CKD (OR: 1.17, 95% CI: 1.01–1.36, *p* = 0.04) after adjusting for multiple covariates. The cut-off point of TG/HDL-C as a predictor of CKD was 2.54 with an area under the ROC curve of 0.61 (95% CI: 0.53–0.68). There was a significant positive correlation between TG/HDL-C and several cardiovascular disease risk factors, including obesity indices. The TG/HDL-C ratio was significantly associated with the risk of CKD and demonstrated predictive ability for CKD in the middle-aged and elderly population. Further studies on its application in clinical settings are warranted.

## Introduction

Chronic kidney disease (CKD) is a major global health issue that increases the risk of both end-stage renal disease (ESRD) and cardiovascular disease (CVD) as well as other complications (1, 2). Globally, the estimated prevalence of CKD is 13.4% (11.7–15.1%), and the number of patients with ESRD is estimated as 4.902–7.083 million (3). Therefore, early screening and managing the risk factors associated with CKD are crucial to preventing disease occurrence.

Obesity has become a global epidemic, and its prevalence has been projected to increase by 40% in the next decade. This increasing prevalence has implications for the risk of diabetes, CVD, and CKD. A high body mass index (BMI) is one of the strongest risk factors for new-onset CKD (4). Obesity is also a risk factor for metabolic syndrome and is closely related to dyslipidemia (5). In Taiwan, according to 2005–2008 data, the prevalence of being overweight and obese among adults was 44.1% and was significantly higher than that in Japan, South Korea, and other neighboring Asian countries (6, 7). Obesity has been reported to be associated with the triglyceride/high-density lipoprotein cholesterol (TG/HDL-C) ratio and CKD in addition to dyslipidemia (8, 9).

Dyslipidemia, defined as having elevated levels of triglyceride (TG), total cholesterol, and low-density lipoprotein-cholesterol (LDL-C) or low levels of high-density lipoprotein-cholesterol (HDL-C), is an important risk factor for CVD and CKD (10, 11). In individuals with dyslipidemia, it has been found that higher TG/HDL-C could increase the risk of CVD by affecting the vessel wall and causing insulin resistance (12). Several studies have also shown that TG/HDL-C is closely associated with the incidence or risk of type 2 diabetes (13), CVD (13–15), coronary heart disease (14, 16), cerebrovascular disease (14), hypertension (HTN) (17), and albuminuria (14). In addition, TG/HDL-C is a marker of increased atherosclerotic extension and may be used to identify subjects with a higher cardiovascular risk profile (15). As glomerulosclerosis and atherosclerosis share a similar pathogenetic process, it is logical to infer that high TG/HDL-C may also be associated with a high risk of CKD development and progression. The role of obesity-related parameters, such as the TG/HDL-C ratio, is becoming increasingly important; however, there is limited information on the ability of the TG/HDL-C ratio in predicting CKD. Therefore, this study attempted to investigate the relationship between TG/HDL-C and CKD in Taiwanese adults.

The CKD prevalence in Taiwan is higher than the global prevalence. A large prospective cohort study in Taiwan reported an estimated prevalence of CKD of 15.46% (18). Furthermore, the prevalence of being overweight and obese among adults is higher in Taiwan than in other neighboring Asian countries (6), and the age-adjusted prevalence of hyperlipidemia in Taiwan is 9.7% (19). Therefore, further investigation of obesity-related parameters and CKD in Taiwan is necessary. In this cross-sectional study, we analyzed the association between the TG/HDL-C ratio as an obesity-related parameter and CKD in the middle-aged and elderly Taiwanese population.

## Materials and methods

### Study design and study population

This was a cross-sectional, community-based study. The participants for this study were recruited from a community health promotion project of Linkou Chang Gung Memorial Hospital between January and October 2014 in Guishan District, Taoyuan City. Initially, we recruited 619 volunteers aged 50 years and above in a consecutive manner through poster promotion or notification from the community office from eight randomized clusters of 28 villages in the Guishan District. Participants were excluded if they (1) had functional disability, (2) refused to participate or could not finish all examinations and the interview, and (3) had recent cardiovascular diseases. A total of 400 participants, including 141 males and 259 females, were eligible for the study. A sample size of 352 can achieve 90% power using two-tail(s), odds ratio = 2.0, probability of null hypothesis = 0.15, alpha error = 0.05, power = 0.9, and *R*^2^ for other confounding factors = 0.5 (20). Therefore, the sample size of 400 subjects implied sufficient statistical power. The project was approved by the Institutional Review Board of Linkou Chang Gung Memorial Hospital, and all participants provided written informed consent before enrollment.

### Data collection and parameter measurements

There were three parts of data collection, including anthropometric measurements, laboratory examination, and a structured questionnaire. For anthropometric measurements, blood pressure (BP), heart rate, BMI, and waist circumference (WC) were recorded. Systolic blood pressure (SBP), diastolic blood pressure (DBP), and heart rate were checked at least twice after 5 min of rest on a chair. BMI was calculated as weight (kg) divided by height squared (m^2^). WC was measured at the mid-point between the lower border of the rib cage and the upper iliac crest on the mid-axillary line. Laboratory examination was performed with blood and urine sampling, and the tests included serum creatinine, fasting plasma glucose (FPG), HDL-C, LDL-C, TG, TG/HDL-C, insulin, and urine albumin/creatinine ratio (ACR). Venous blood samples were collected after overnight fasting for at least 12 h. Urine specimens were obtained in the morning, and sampling was scheduled to avoid menstrual periods. A standardized biochemistry analyzer (ABBOTT Alinity i-eries system, Germany) was used for determination of plasma insulin with Alinity i Insulin Reagent Kit, and the rest of laboratory tests were determined by Hitachi LST008 (Japan) with the kits from Shino, MeDiPro, FUJIFILM Wako Pure Chemical, SEKISUI, and Hitachi Chemical. Each participant completed a structured questionnaire during a face-to-face interview by trained interviewers, and the questionnaire included personal information, smoking/drinking habits, past medical history, and current medication use.

### Definitions of CKD

Chronic kidney disease is defined as the presence of kidney damage (urine ACR ≥30 mg/g) or decreased renal function with an estimated glomerular filtration rate (eGFR) < 60 ml/min/1.73 m^2^ according to the 2005 guidelines of Kidney Disease: Improving Global Outcomes (KDIGO) (21). The eGFR was calculated using the 2009 Chronic Kidney Disease Epidemiology Collaboration (CKD-EPI) equation:


$$\begin{array}{l} \text{eGFR}=141\;\times \min\;{\left(\text{Scr }/\kappa ,\;1\right)}^{\alpha }\;\times \max\;{\left(\text{Scr }/\kappa ,\;1\right)}^{-1.209}\\ \;\times 0.993^{\text{Age}}\times 1.018\;\left[\text{if female}\right]\times 1.159\;\left[\text{if black}\right] \end{array}$$

Where Scr is serum creatinine in mg/dl, κ is 0.7 for females and 0.9 for males, α is −0.329 for females and −0.411 for males, min indicates the minimum of Scr /κ or 1, and max indicates the maximum of Scr/κ or 1 (22).

### Statistical analysis

The G^*^power 3.1 software was used for sample size determination. General characteristics are expressed as mean ± standard deviation (SD) for continuous variables and number (%) for categorical variables. The *p*-values were determined from an independent two-sample *t*-test and chi-square test for continuous and categorical variables, respectively. Pearson's correlation test was used to determine the correlation between TG/HDL-C and CVD risk factors. Multiple logistic regression models were developed to investigate the independent variables of TG/HDL-C associated with CKD. We used the area under the receiver operating characteristic (ROC) curve (AUC) and 95% confidence interval (CI) to assess the discriminatory power of TG/HDL-C in predicting the risk of CKD. All statistical analyses were performed using SPSS for Windows, SPSS version 27.0.1.0 (SPSS Inc., Chicago, IL). A *p*-value of < 0.05 was considered to be significant.

## Results

A total of 400 participants aged 50 years or older (mean age: 64.47 ± 8.45 years, 35.3% males) were enrolled in this study. The study population was divided into two groups according to the presence of CKD as shown in Table 1. Of the total participants, 81 (20.3%) of them had CKD, and 141 of them were male participants. The mean eGFR of the total participants was 112.97 ± 33.43 ml/min/1.73 m^2^. The mean eGFR of the CKD group was 96.25 ± 46.29 ml/min/1.73 m^2^, and the mean eGFR of the non-CKD group was 117.21 ± 27.82 ml/min/1.73 m^2^ (*p* < 0.001). The TG/HDL-C ratios of the CKD and non-CKD groups were 3.37 ± 2.72 and 2.35 ± 1.66, respectively (*p* = 0.002), suggesting that the TG/HDL-C ratio of the CKD group was significantly higher than that of the non-CKD group. Furthermore, there were significant differences in age, SBP, DBP, WC, creatinine, FPG, HDL-C, insulin, LDL-C, and TG. The proportion of those who were current smokers and alcohol consumers was lower in the CKD group than in the non-CKD group. It is possible that they quit smoking and drinking alcohol after being diagnosed with CKD. Analysis of the past medical history of the participants revealed that the prevalence of HTN and diabetes mellitus (DM) but not hyperlipidemia was significantly higher in the CKD group than in the non-CKD group. Analysis of the current medication use of the participants revealed that the prevalence of Chinese herb use was significantly higher in the non-CKD group than in the CKD group; however, there was no significant difference in the prevalence of analgesic use.

**Table 1:** General characteristics of the study population according to the presence of chronic kidney disease (CKD).

**Variables**	**Chronic kidney disease**			
	**Total**	**Yes**	**No**	***p*-Value**
	**(*n* = 400)**	**(*n* = 81)**	**(*n* = 319)**	
Age (year)	64.47 ± 8.45	66.67 ± 9.71	63.91 ± 8.02	0.02
SBP (mmHg)	129.50 ± 16.71	135.38 ± 16.51	128.01 ± 16.46	< 0.001
DBP (mmHg)	76.93 ± 11.36	79.99 ± 13.42	76.15 ± 10.66	0.01
BMI (kg/m^2^)	24.55 ± 3.57	25.10 ± 3.93	24.41 ± 3.46	0.12
Waist circumference (cm)	85.07 ± 9.68	87.11 ± 10.63	84.55 ± 9.37	0.03
Creatinine (mg/dl)	0.78 ± 0.43	1.06 ± 0.83	0.70 ± 0.17	< 0.001
eGFR (ml/min/1.73 m^2^)	112.97 ± 33.43	96.25 ± 46.29	117.21 ± 27.82	< 0.001
FPG (mg/dl)	96.23 ± 25.73	105.31 ± 40.20	93.93 ± 19.95	0.02
HDL-C (mg/dl)	54.43 ± 13.93	51.04 ± 15.16	55.29 ± 13.49	0.01
Insulin (μU/ml)	9.21 ± 25.98	15.99 ± 55.79	7.48 ± 6.87	0.01
LDL-C (mg/dl)	118.37 ± 32.11	110.68 ± 29.16	120.32 ± 32.57	0.02
TG (mg/dl)	122.07 ± 65.97	145.95 ± 87.39	116.01 ± 57.94	0.004
TG/HDL-C	2.55 ± 1.96	3.37 ± 2.72	2.35 ± 1.66	0.002
Current smoker, *n* (%)	43 (10.8)	8 (9.9)	35 (11.0)	0.78
Alcohol consumption, *n* (%)	75 (18.8)	7 (8.6)	68 (21.3)	0.01
Male, *n* (%)	141 (35.3)	31 (38.3)	110 (34.5)	0.52
ACR ≥30 mg/g, *n* (%)	75 (18.8)	75 (92.6)	0 (0.0)	< 0.001
HTN, *n* (%)	201 (50.3)	59 (72.8)	142 (44.5)	< 0.001
DM, *n* (%)	79 (19.8)	30 (37.0)	49 (15.4)	< 0.001
Hyperlipidemia, *n* (%)	260 (65.0)	57 (70.4)	203 (63.6)	0.26
Chinese herb use, *n* (%)	129 (32.3)	18 (22.2)	111 (34.8)	0.03
Analgesic use, *n* (%)	32 (8.0)	9 (11.1)	23 (7.2)	0.25

Clinical characteristics are expressed as mean ± SD for continuous variables and n (%) for categorical variables. p-value were derived from independent two-sample t-test for continuous variables and chi-square test for categorical variables.

SBP, systolic blood pressure; DBP, diastolic blood pressure; BMI, body mass index; eGFR, estimated glomerular filtration rate; FPG, fasting plasma glucose; HDL-C, high-density lipoprotein-cholesterol; LDL-C, low-density lipoprotein-cholesterol; TG, triglyceride; ACR, albumin to creatinine ratio; HTN, hypertension; DM, diabetes mellitus.

**Figure 1:**
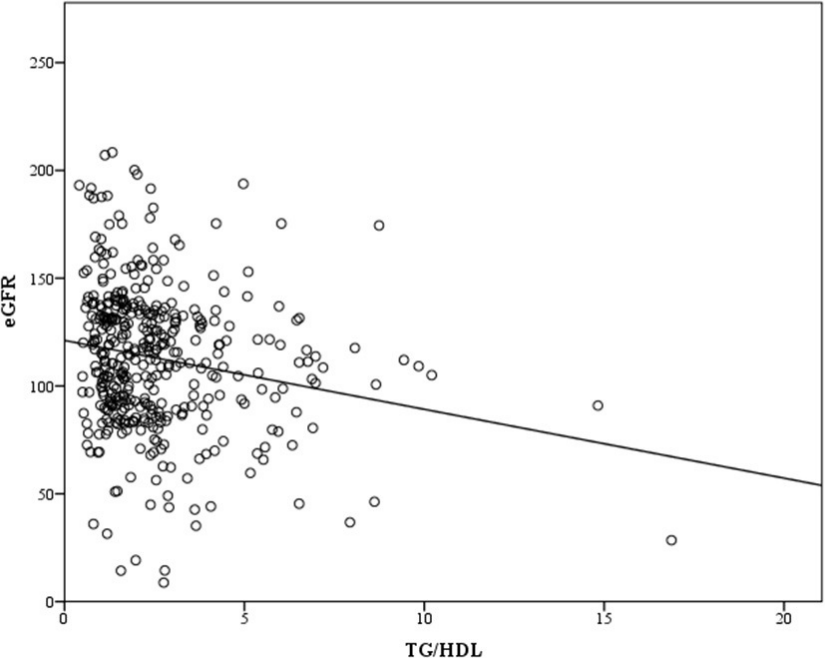
Representation of the correlation analysis: there was a trend toward a negative correlation between triglyceride/high-density lipoprotein-cholesterol (TG/HDL-C) and estimated glomerular filtration rate (eGFR) levels.

The correlation between TG/HDL-C and CVD risk factors was evaluated as shown in Table 2. After adjusting for age, TG/HDL-C was significantly positively correlated with SBP, DBP, BMI, WC, and FPG but not LDL-C. Figure 1 shows the correlation between TG/HDL-C and eGFR. The eGFR level was negatively correlated (*p* < 0.001, *R*^2^ = 0.035) with the TG/HDL-C ratio.

**Table 2:** Correlation between triglyceride/high-density lipoprotein-cholesterol (TG/HDL-C) and cardiovascular disease risk factors.

**Variables**	**TG/HDL-C (*****n*** = **400)**			
	**Unadjusted**		**Adjusted for age**	
	**Pearson's coefficient**	***p*-Value**	**Pearson's coefficient**	***p*-Value**
Age (year)	0.05	0.35	NA	NA
SBP (mmHg)	0.17	0.001	0.16	0.001
DBP (mmHg)	0.15	0.003	0.17	0.00
BMI (kg/m^2^)	0.27	< 0.001	0.27	< 0.001
Waist circumference (cm)	0.33	< 0.001	0.33	< 0.001
FPG (mg/dl)	0.34	< 0.001	0.34	< 0.001
LDL-C (mg/dl)	−0.10	0.06	−0.09	0.07

SBP, systolic blood pressure; DBP, diastolic blood pressure; BMI, body mass index; FPG, fasting plasma glucose; HDL-C, high-density lipoprotein-cholesterol; LDL-C, low-density lipoprotein-cholesterol; TG, triglyceride.

The results of the multiple logistic regression model analysis are shown in Table 3. Model 1 was adjusted for sex, model 2 was adjusted for sex, age, and BMI, and model 3 was adjusted for sex, age, BMI, smoking status, past medical history (including DM, HTN, and hyperlipidemia), Chinese herb use, analgesic use, and insulin levels. After adjusting for these confounding factors, TG/HDL-C was still significantly associated with CKD (OR: 1.17, 95% CI: 1.01–1.36, *p* = 0.04).

**Table 3:** Multiple logistic regression on the factors related to chronic kidney disease (CKD) in the screened population (*n* = 400).

**Variables**	**Odds ratio**	**CKD versus non-CKD 95% CI**	***p*-Value**
**Model 1**			
TG/HDL-C	1.25	(1.11–1.41)	< 0.001
Sex (male vs. female)	0.99	(0.59–1.68)	0.97
**Model 2**			
TG/HDL-C	1.24	(1.09–1.40)	0.001
Sex (male vs. female)	0.90	(0.53–1.54)	0.71
Age (year)	1.04	(1.01–1.07)	0.01
BMI (kg/m^2^)	1.03	(0.96–1.11)	0.44
Model 3
TG/HDL	1.17	(1.01–1.36)	0.04
Sex (men vs. women)	1.04	(0.58–1.87)	0.89
Age (year)	1.02	(0.99–1.06)	0.16
BMI (kg/m^2^)	0.99	(0.91–1.07)	0.75
Smoking (yes vs. no)	0.64	(0.24–1.74)	0.38
DM (yes vs. no)	2.48	(1.39–4.43)	0.00
HTN (yes vs. no)	2.22	(1.21–4.07)	0.01
Hyperlipdemia (yes vs. no)	1.01	(0.54–1.88)	0.97
Chinese herb use (yes vs. no)	0.97	(0.38–2.49)	0.94
Analgesics use (yes vs. no)	0.70	(0.38–1.28)	0.25
Insulin (μU/ml)	1.01	(0.98–1.05)	0.40

BMI, body mass index; HDL-C, high-density lipoprotein-cholesterol; TG, triglyceride; HTN, hypertension; DM, diabetes mellitus; CI, confidence interval.

**Figure 2:**
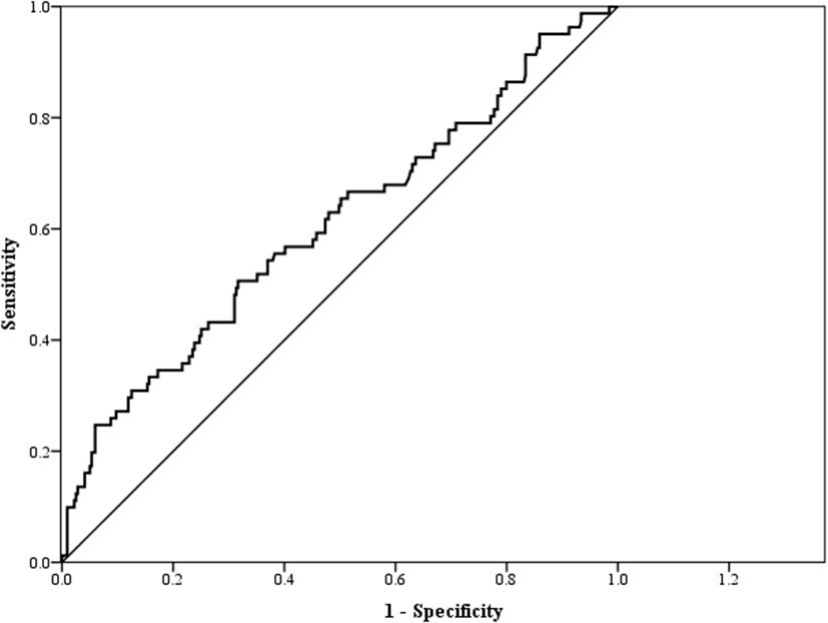
Receiver operating characteristic (ROC) curve for triglyceride/high-density lipoprotein-cholesterol (TG/HDL-C) as a predictor of chronic kidney disease (CKD).

Table 4 and Figure 2 show the AUC score (and 95% CI), sensitivity, and specificity with the optimized cut-off point of TG/HDL-C for predicting CKD. The cut-off point of TG/HDL-C as a predictor of CKD was 2.54 with an AUC of 0.61 (95% CI: 0.53–0.68, *p* = 0.003). Therefore, TG/HDL-C was significantly capable of predicting CKD.

**Table 4:** Area under the receiver operating characteristic (ROC) curve (AUC), sensitivity, and specificity by the optimized cut-off point for triglyceride/high-density lipoprotein-cholesterol (TG/HDL-C) in predicting chronic kidney disease (CKD).

**Variables**	**AUC (95% CI)**	***p*-Value**	**Cut-off point**	**Sensitivity**	**Specificity**
TG/HDL-C	0.61 (0.53–0.68)	0.003	2.54	0.51	0.68

HDL-C, high-density lipoprotein-cholesterol; TG, triglyceride; CKD, chronic kidney disease; ROC curve, receiver operating characteristic curve; CI, confidence interval.

## Discussion

This cross-sectional study of a Taiwanese population demonstrated a significant association of the TG/HDL-C ratio with CKD, the predictive ability of TG/HDL-C for CKD, and a significant correlation between TG/HDL-C and eGFR as well as cardiovascular risk factors.

Previous studies have reported the association of TG/HDL-C with CKD (14, 23, 24), which is consistent with our findings. To the best of our knowledge, this study is the first to associate TG/HDL-C with CKD in the middle-aged and elderly Taiwanese populations, and there is limited information on the ability of the TG/HDL-C ratio in predicting CKD. The CKD prevalence in our study (20.3%) was higher than that in a large Taiwanese cohort of a previous study (18), which may be partly attributed to the older age of the participants in the present study.

Dyslipidemia in CKD is largely associated with increased TG, decreased HDL-C, and varying levels of LDL-C (25). TG-rich lipoproteins, including very-low-density lipoprotein (VLDL), chylomicrons, and chylomicron remnants, start to increase in the early stages of CKD because of suppressed lipoprotein lipase (LPL)-mediated hydrolysis of VLDL and chylomicrons and an excess of lipase inhibitors such as apoC-III and pre-beta-HDL in uremic plasma (25, 26); these TG-rich lipoprotein remnants contribute to the initiation and progression of atherosclerosis (27). On the other hand, the cardiovascular protective effect of HDL-C has been attributed to its ability to act as both a cholesterol acceptor and a cholesterol carrier in the reverse cholesterol transport (RCT) pathway, including cholesterol delivery to the liver. An observational study estimated that the cardiovascular risk decreases by around 2–3% per 1 mg/dl increase in HDL-C (28). Patients with advanced kidney disease have a reduced level of lecithin cholesterol ester transfer protein, thus causing the failure of HDL maturation, which results in increased pre-beta HDL and TG-rich HDL with decreased effective anti-oxidation activity (29). Furthermore, CKD downregulates hepatic apoA-I gene expression and hepatic LCAT mRNA expression, resulting in decreased HDL and impaired HDL-mediated cholesterol uptake from vascular tissues (30, 31). Previous studies have found that a high TG/HDL-C ratio is significantly associated with an increased incidence or risk of CVD (13–15). A prospective cohort study showed that an elevated TG/HDL-C ratio is strongly associated with an increased risk of major adverse cardiac events and is an independent predictor of long-term all-cause mortality (32). In addition, a retrospective cohort study (33) and several cross-sectional studies (23, 24) have revealed the relationship between TG/HDL-C and CKD. In the present study involving the middle-aged and elderly Taiwanese population, we obtained similar results.

In our study, we observed a significant positive correlation of TG/HDL-C with several CVD risk factors, such as FPG, BP, BMI, and WC, after adjusting for age. A retrospective cohort study using data from the Stanford Translational Research Integrated Database Environment confirmed that high TG/HDL-C (>2.5 in females or >3.5 in males) was significantly associated with an increased incidence of type 2 diabetes (13). Furthermore, a Spanish cohort study found that in men, those in the top quintile of the TG/HDL-C ratio were two times more likely than those in the bottom quintile to develop HTN, suggesting an association between TG/HDL-C and incident HTN (17). The ELSA-Brasil cohort study showed that the defined TG/HDL-C cut-offs (males: 2.6; females: 1.7) are reliable with good clinical applicability for detecting cardiometabolic conditions, including obesity and increased WC, in a multiethnic population (8). Overall, the findings of our study are consistent with those of other studies.

Several limitations in our study should be considered. First, the cross-sectional design limited our ability to infer causality between TG/HDL-C and CKD. Second, the participants were enrolled voluntarily from a northern community in Taiwan, which may result in possible healthy volunteer bias and uncertainty of the external validity of the findings. Volunteer bias could potentially influence the prevalence of CKD and the associated risk factors, and those who require substantial care or assistance due to severe CKD or associated risk factors might not be included in the study. Therefore, this population was not nationally representative. Third, acute kidney injury could not be excluded from our study. Finally, there were some known risk factors of CKD that were not considered in the study, which might also be confounding factors, such as structural or functional kidney abnormalities and certain environmental or occupational exposures.

## Conclusions

In our study, we observed the significant positive correlation of TG/HDL-C with several CVD risk factors, including obesity indices. Furthermore, we found that TG/HDL-C was negatively correlated with eGFR and significantly associated with the risk of CKD after adjusting for multiple confounding factors. Finally, the findings demonstrated the predictive ability of TG/HDL-C for CKD.

## Data availability statement

The original contributions presented in the study are included in the article/supplementary material, further inquiries can be directed to the corresponding author.

## Ethics statement

The studies involving human participants were reviewed and approved by Institutional Review Board of Linkou Chang Gung Memorial Hospital. The patients/participants provided their written informed consent to participate in this study.

## Author contributions

J-YC planed and conceptualized the work, performed data collection and analysis, and revised the draft. I-JC wrote the original draft. J-YC, L-TH, and T-WL supervised the work. All authors contributed to the article and approved the submitted version.

## Funding

This study was supported by Chang Gung Memorial Hospital (grants CORPG3C0171~3C0172, CZRPG3C0053, CORPG3G0021, CORPG3G0022, and CORPG3G0023).

## Conflict of interest

The authors declare that the research was conducted in the absence of any commercial or financial relationships that could be construed as a potential conflict of interest.

## Publisher's note

All claims expressed in this article are solely those of the authors and do not necessarily represent those of their affiliated organizations, or those of the publisher, the editors and the reviewers. Any product that may be evaluated in this article, or claim that may be made by its manufacturer, is not guaranteed or endorsed by the publisher.
